# Quinpirole ameliorates nigral dopaminergic neuron damage in Parkinson’s disease mouse model through activating GHS-R1a/D_2_R heterodimers

**DOI:** 10.1038/s41401-023-01063-0

**Published:** 2023-03-10

**Authors:** Ting-ting Tang, Ming-xia Bi, Mei-ning Diao, Xiao-yi Zhang, Ling Chen, Xue Xiao, Qian Jiao, Xi Chen, Chun-ling Yan, Xi-xun Du, Hong Jiang

**Affiliations:** 1 Department of Physiology, Shandong Provincial Key Laboratory of Pathogenesis and Prevention of Neurological Disorders and State Key Disciplines: Physiology, School of Basic Medicine, Qingdao University, Qingdao, 266021 China; 2 University of Health and Rehabilitation Sciences, Qingdao, 266000 China

**Keywords:** Parkinson’s disease, growth hormone secretagogue receptor 1a, dopamine type 2 receptor, heterodimers, quinpirole, CREB signaling pathway

## Abstract

Growth hormone secretagogue receptor 1a (GHS-R1a) is an important G protein-coupled receptor (GPCR) that regulates a variety of functions by binding to ghrelin. It has been shown that the dimerization of GHS-R1a with other receptors also affects ingestion, energy metabolism, learning and memory. Dopamine type 2 receptor (D_2_R) is a GPCR mainly distributed in the ventral tegmental area (VTA), substantia nigra (SN), striatum and other brain regions. In this study we investigated the existence and function of GHS-R1a/D_2_R heterodimers in nigral dopaminergic neurons in Parkinson’s disease (PD) models in vitro and in vivo. By conducting immunofluorescence staining, FRET and BRET analyses, we confirmed that GHS-R1a and D_2_R could form heterodimers in PC-12 cells and in the nigral dopaminergic neurons of wild-type mice. This process was inhibited by MPP^+^ or MPTP treatment. Application of QNP (10 μM) alone significantly increased the viability of MPP^+^-treated PC-12 cells, and administration of quinpirole (QNP, 1 mg/kg, i.p. once before and twice after MPTP injection) significantly alleviated motor deficits in MPTP-induced PD mice model; the beneficial effects of QNP were abolished by GHS-R1a knockdown. We revealed that the GHS-R1a/D_2_R heterodimers could increase the protein levels of tyrosine hydroxylase in the SN of MPTP-induced PD mice model through the cAMP response element binding protein (CREB) signaling pathway, ultimately promoting dopamine synthesis and release. These results demonstrate a protective role for GHS-R1a/D_2_R heterodimers in dopaminergic neurons, providing evidence for the involvement of GHS-R1a in PD pathogenesis independent of ghrelin.

## Introduction

Parkinson’s disease (PD) is a chronic disease of the central nervous system that affects the mobility of patients. It mostly occurs in middle-aged and elderly people [1, 2]. The main pathological changes are the loss of dopaminergic neurons in the substantia nigra pars compacta, which further reduces the release of dopamine (DA) in the striatum and leads to movement disorders [3, 4]. Growth hormone secretagogue receptor 1a (GHS-R1a) is a typical G-protein-coupled receptor (GPCR) that is widely distributed in the hippocampus, substantia nigra (SN), hypothalamus, pituitary and ventral tegmental area (VTA) [5, 6]. In our previous study, GHS-R1a was found to increase the excitability of nigral dopaminergic neurons by inhibiting voltage-gated potassium channels through the activation of the PLC-PKC pathway [7]. A study also demonstrated that motor coordination dysfunction could be observed when a selective GHS-R1a antagonist was stereotactically injected into the SN of normal control mice [8]. Downregulation of GHS-R1a could also lead to motor coordination dysfunction [8] and mood disorders [9]. Conversely, a GHS-R1a agonist was found to mitigate behavioral dysfunction in spontaneously hyperactive mice and cocaine-exposed mice, thereby modulating abnormal DA-mediated behavior [10].

In addition, there is growing evidence that GHS-R1a can participate in a variety of physiological activities in vivo by forming heterodimers with other receptors in the absence of ghrelin, exerting a ligand-independent constitutive effect [11–15]. In the periphery, GHS-R1a can form heterodimers with melanocortin 3 receptor [16, 17], 5-hydroxytryptamine 2c receptor [18, 19], orexin 1 receptor [20, 21] and G protein-coupled receptor 83 [22] to regulate food intake and energy metabolism. In the dentate gyrus of the hippocampus, GHS-R1a can form heterodimers with dopamine type 1 receptor (D_1_R) to regulate synaptic plasticity through activation of the Gαq-PLC-IP3-Ca^2+^ pathway by a D1R agonist [23]. Dopamine type 2 receptor (D_2_R) is a GPCR with seven transmembrane domains [24, 25] that is mainly distributed in the SN, nucleus accumbens, striatum, VTA, olfactory tubercle and hypothalamus [26]. Previous studies showed that *D*_*2*_*R*^*−/−*^ rats exhibited PD-like behavioral impairment, such as spontaneous motor dysfunction [27], decreased motion initiation frequency [27] and spontaneous muscle tone [28] and impaired motor skill learning [27]. Given the roles of GHS-R1a and D_2_R in regulating the function of dopaminergic neurons, it is of vital importance to clarify whether endogenous GHS-R1a and D_2_R could form heterodimers in the SN and the role of these heterodimers in dopaminergic neurons in PD.

cAMP response element binding protein (CREB) is a broad transcriptional regulatory factor that regulates the expression of brain-derived neurotrophic factor, activating transcription factor-3, transcription 3, c-Fos, cAMP response element modulator, cyclin A and other genes [29–34]. It plays an important role in synaptic plasticity, learning and memory, and neuronal development [34–38]. CREB can be phosphorylated upstream of Ca^2+^/calmodulin (CaM) [39], promoting the growth of dorsal root ganglion neurons by upregulating brain-derived neurotrophic factor [40] and alleviating the symptoms of Alzheimer’s disease in mice [41]. As two key factors in the synthesis and release of DA [42–45], tyrosine hydroxylase (TH) and vesicle monoamine transporter 2 (VMAT2) are both downstream targets of CREB [46–48]. In addition, the activity of CaM protein is decreased in 1-methyl-4-phenyl-1,2,3,6-tetrahydropyridine (MPTP)-induced PD mice model, resulting in cognitive deficits and learning disabilities [49, 50]. However, it is not clear whether the activation of GHS-R1a/D_2_R heterodimers affects the expression of TH and VMAT2 via CREB phosphorylation, thus regulating DA synthesis and metabolism.

In the present study, the existence of GHS-R1a/D_2_R heterodimers was confirmed in dopaminergic cells and neurons. Using MPTP-induced PD mice model, we aimed to investigate whether activation of the GHS-R1a/D_2_R heterodimers affects the synthesis and release of DA. To explore the underlying mechanism, we assessed the protein levels of CaM, CREB and downstream targets of TH and VMAT2. We aimed to elucidate the potential molecular mechanisms of GHS-R1a/D_2_R heterodimers on PD pathogenesis to provide a new drug target for PD treatment.

## Materials and methods

### Animals and treatment

GHS-R1a knockout (*Ghsr*^−/−^) mice were ordered from the Shanghai Model Organisms Center. Male *Ghsr*^−/−^ mice and their littermate wild-type (WT) mice were used in this experiment. Fifteen to sixteen-week-old mice weighing (25 ± 2) g were housed on a 12 h light/dark cycle at a room temperature of (22 ± 2) °C to mimic natural conditions. The mice were given food and purified water ad libitum. Animal experiments were carried out according to the National Institutes of Health Guidelines for the Care and Use of Laboratory Animals. All protocols were approved by the Animal Ethics Committee of Qingdao University.

Adult mice were intraperitoneally injected with 0.5 or 1 mg/kg quinpirole (QNP) (TOCRIS). Behavioral tests were performed at 0.5, 1, 2, 4 and 6 h after injection, and brain tissues were collected. Then, the microdialysis experiment was carried out first, and 1 day after recovery, *Ghsr*^−/−^ and WT mice were intraperitoneally injected with 1 mg/kg QNP or normal saline (NS), 4 h before MPTP (Sigma) injection (Fig. 4a). Then, MPTP (20 mg/kg) was intraperitoneally injected 4 times at an interval of 2 h for acute MPTP treatment. Finally, the mice were injected with QNP (1 mg/kg) twice at an interval of 4 h. On the fourth and fifth days, the mice were subjected to behavioral tests and a microdialysis study. The animals were sacrificed, and their brains were harvested on the sixth day.

### Cell culture and transfection

PC-12 cells were purchased from the National Infrastructure of Cell Line Resource (Shanghai, China) and cultured in RPMI-1640 medium with 10% fetal bovine serum (TransGen Biotech, China) and 1% penicillin‒streptomycin at 37 °C in 5% CO_2_. Cell transfection was performed by Lipofectamine 2000 (Invitrogen, USA) reagent according to the manufacturer’s protocol.

### Plasmids and viruses

PC-12 cells were transfected with the pcDNA3.1-D_2_R, pcDNA3.1-GHS-R1a, or pcDNA3.1-D_2_R/pcDNA3.1-GHS-R1a plasmid. The D_2_R and GHS-R1a plasmids were purchased from OBiO Technology (Shanghai, China). To establish stable cell lines, PC-12 cells were transfected with shRNA-GHS-R1a, which was purchased from OBiO Technology (Shanghai, China).

### Immunofluorescence staining

Brain tissues were trimmed, and tissues containing the substantia nigra pars compacta were sectioned into 20 μm slices (Leica, Germany). Fifty frozen sections from each mouse were divided into four sets. One set was randomly selected for immunofluorescence staining to observe the colocalization of GHS-R1a and D_2_R. Brain slices and cells were blocked with 10% goat serum and incubated with mouse anti-GHS-R1a (Absin, 1: 50) and rabbit anti-D_2_R (Santa Cruz, 1: 50) overnight at 4 °C. The cells or tissues were then washed with PBS and incubated with Alexa Fluor® 555-conjugated goat anti-mouse IgG (H + L) (1:500), Alexa Fluor® 488-conjugated goat anti-rabbit IgG (H + L) (1:500) and Alexa Fluor® 555-conjugated goat anti-rabbit IgG (H + L) (1:500) secondary antibodies for 1 h at room temperature, and DAPI was added for 5 min before mounting with antifade solution. Images were acquired using immunofluorescence microscopy (Leica, Germany).

### Western blotting

The SN was precisely dissected out, and the tissues were incubated with RIPA lysis buffer (CWBIO, China) containing protease inhibitors and lysed for 30 min on ice after being fully ground. After centrifugation at 12,000 rpm for 20 min at 4 °C, the supernatant was removed for determination of the protein concentration by a Bradford assay kit (CWBIO, China). Twenty milligrams of total protein were loaded on a 10% SDS polyacrylamide gel and then transferred onto a 0.45 mm PVDF membrane (300 mV, 90 min). After blocking with 5% nonfat milk at room temperature for 2 h, the membranes were incubated with anti-rabbit TH antibody (Abcam, 1:4000), anti-rabbit CaM antibody (Cell Signaling Technology, 1:1000), anti-rabbit p-CREB antibody (Cell Signaling Technology, 1:1000), anti-rabbit CREB antibody (Cell Signaling Technology, 1:1000), anti-rabbit GHS-R1a antibody (Phoenix Pharmaceuticals, 1:1000), and anti-rabbit GAPDH antibody (Cell Signaling Technology, 1:10,000) overnight at 4 °C. The membranes were then incubated with anti-rabbit secondary antibodies conjugated to horseradish peroxidase at 1:10,000 for 2 h at room temperature. Cross-reactivity was visualized using ECL Western blotting detection reagents and analyzed by scanning densitometry using an Amersham Image Quant 800 imaging system (Cytiva, USA).

### Real-time polymerase chain reaction (RT-PCR)

Total RNA was isolated from the mouse SN using TRIzol reagent (Invitrogen, USA) according to the manufacturer’s instructions. One microgram of RNA was transcribed into cDNA in a 20 μl reaction with a reverse-transcription kit (Vazyme, China). RT-PCR was carried out using SYBR Green Master Mix (Vazyme, China). The expression of each gene was normalized to that of GAPDH. The primer sequences were as follows: VMAT2: F = 5′-AAGAGAGGGGTAACGCCAT-3′, R = 5′-AGCAAGCACCAGGAAAGGA-3′; GAPDH: F = 5′-CAAATTCCATGGCACCGTCA-3′, R = 5′-ATCGCCCCACTTGATTTTGG-3′.

### Proximity ligation assay (PLA)

PC-12 cells were grown and fixed with 4% paraformaldehyde prepared in PBS on 6-well tissue culture slides. To visualize receptor interactions, slides or brain slices were blocked with blocking solution and incubated with mouse anti-GHS-R1a (Absin, 1:25) and rabbit anti-D_2_R (Thermo Fisher Scientific, 1:50) antibodies at 4 °C overnight in a humidified chamber. The slides or brain slices were then washed and incubated with secondary anti-rabbit/goat antibodies conjugated to PLUS and MINUS Duolink II PLA (1:5) at 37 °C for 1 h. The slides or brain slices were washed again and then incubated with ligation-ligase solution (30 min at 37 °C), followed by incubation with amplification-polymerase solution (90 min at 37 °C). PLA signals (594/624 nm) were identified as fluorescent spots under a fluorescence microscope (Leica, Germany) at room temperature and quantified with ImageJ software.

### Bioluminescence resonance energy transfer (BRET)

PC-12 cells were transiently cotransfected with vectors encoding Rluc fusion (GHS-R1a-Rluc) or EGFP fusion (D_2_R-EGFP) proteins. Twenty-four hours after transfection, the cells were washed twice, detached, and transferred (10^5^ cells/well) to a 96-well microplate (Corning 3600, white opaque plate) containing HEPES-buffered phenol red-free medium (Invitrogen, Life Technologies) for 24 h. The cells were then washed twice and resuspended in D-PBS (PBS containing 0.5 mM MgCl_2_ and 0.1% (w/v) glucose). Coelenterazine H substrate was added to a final concentration of 5 μM. Analysis was carried out immediately at 37 °C using a Tristar LB941 plate reader (Germany).

### Fluorescence resonance energy transfer (FRET)

The donor plasmid D_2_R-EYFP and the receptor plasmid GHS-R1a-ECFP were cotransfected into PC-12 cells. FRET signals were detected using a Nikon A1R confocal microscope (Japan), which allowed the sequential integration of light signals detected with two filter settings. The donor channel was set to detect the excitation light of the fluorescent protein EYFP, and the accepter channel was set to detect the emission light of the fluorescent protein ECFP. FRET from the donor channel and the accepter channel was observed.

### Microdialysis

Microdialysis guide cannulas (CMA Microdialysis AB, Sweden) were implanted into the mouse striatum. Mice were anesthetized with isoflurane gas (4% induction and 1.5%–3% maintenance; Abbott Laboratories, USA) prior to being fixed in a stereotaxic frame (RIWARD, China). The skull was adjusted so that bregma and lambda lay in the same horizontal plane to ensure that the skull was level (with ±0.1 mm tolerance), and 2 points equally distant from the midline were marked. The stereotaxic coordinates of the striatum were as follows: anterior = 0.8 mm, lateral = 1.8 mm, ventral = 3.0 mm. A hole was drilled in the skull at these coordinates, and the guide cannula was slowly lowered into the hole. The guide cannula was attached to the skull with super glue and dental cement. Following surgery, the animal was placed in a heated chamber until it awoke and moved normally.

Tubing was connected to each microsyringe. Artificial cerebrospinal fluid consisting of NaCl (147 mM), KCl (2.7 mM), CaCl_2_ (1.2 mM), and MgCl_2_ (0.85 mM) was used. After the mice were connected to the sampling apparatus, the room lights were turned off for the duration of the experiment. The animals were awake and freely moving while connected to the sampling apparatus to avoid any artifacts produced by anesthesia (1 μl/min). After 30 min of stabilization, cerebrospinal fluid was collected by an automated sample collector at a volume of 20 μl per mouse for 20 min, and cerebrospinal fluid DA contents were determined.

### Cell viability measurement

Cell viability was measured using the Cell Counting Kit-8 (CCK-8) assay. PC-12 cells were seeded in 96-well plates, and the culture medium was replaced with 10 μl of CCK-8 solution (Solarbio, China) for 1 h at 37 °C in the dark after treatment. The absorbance at 450 nm was measured using a microplate reader (VICTOR Nivo, PerkinElmer, Finland).

### Transmission electron microscopy

PC-12 cells were fixed with 2.5% glutaraldehyde for 24 h at 4 °C. Then, 1% osmic acid was added for 1 h for dehydration. The cells were embedded and incubated at 60 °C for 24 h. After sectioning, the cells were washed with ddH_2_O, immersed in uranyl acetate and lead citrate solution, and then washed three times with ddH_2_O. Finally, transmission electron microscopy was used for observation.

### Rotarod test

A rotarod apparatus (Med Associates, USA) was used to measure balance and motor coordination. During the training period, the mice were allowed to adapt to the rotarod for 2 min without rotation. Then, the drum was slowly accelerated from a speed of 4–40 rpm for a maximum of 5 min. The latency to fall off the rotarod within this time period was recorded. Each mouse was tested twice at an interval of at least 1 h, and the results were averaged.

### Open field test

The experiment was conducted in a quiet and dark environment. Exploratory activity was measured using an open-field apparatus (42 cm × 42 cm). Each mouse was placed in the center of the open-field apparatus. The center zone was defined as a square 10 cm away from each wall. The distance traveled and time spent in the zone by each animal were recorded for 10 min with a video-recording system (Smart 2.0, Panlab), as described previously.

### High-performance liquid chromatography with electrochemical detection (HPLC-ECD)

After the mice were anesthetized, they were quickly decapitated, and the bilateral striata were quickly removed on ice. Eighty microliters of precooled sample pretreatment A solution (0.4 M perchloric acid) were added to accurately weigh striatal tissue. Then, the tissue was ground and centrifuged at 4 °C for 20 min at 12,000 rpm. Forty microliters of the supernatant were added to 80 µl of ice-cold pretreatment B solution (dipotassium phosphate, 300 mM; EDTA-2Na, 2 mM; citramalic acid-potassium, 20 mM), vortexed, mixed, left on ice for 1 h and centrifuged at 4 °C for 20 min at 12,000 rpm. The samples were filtered 1–2 times before being loaded into the machine. The contents of DA and its metabolites 3,4-dihydroxyphenylacetic acid (DOPAC) and homovanillic acid (HVA) in 100 µl of the supernatant were determined by HPLC-ECD (Waters, USA). The flow rate was set to 0.5–1 µl/min, and each sample was examined for 5–10 min. Each standard was tested first, and its retention time was tested as a qualitative index. A standard curve was drawn with data for standard samples (16, 8, 4, 2, 1, 0.5, 0.25, 0.125 ng/20 µl) to obtain the linear regression equation of each standard, and then the sample concentration was calculated.

### Data analysis

In this experiment, GraphPad Prism 8 software was used to analyze and plot the data. One-/two-way ANOVA was used, and the experimental results are expressed as the mean ± SEM. When comparing means of more than two groups, Dunnett’s test or Student’s Newman–Keul test was used. *P* < 0.05 was considered statistically significant.

## Results

### GHS-R1a and D_2_R formed heterodimers both in vitro and in vivo

**Fig. 1:**
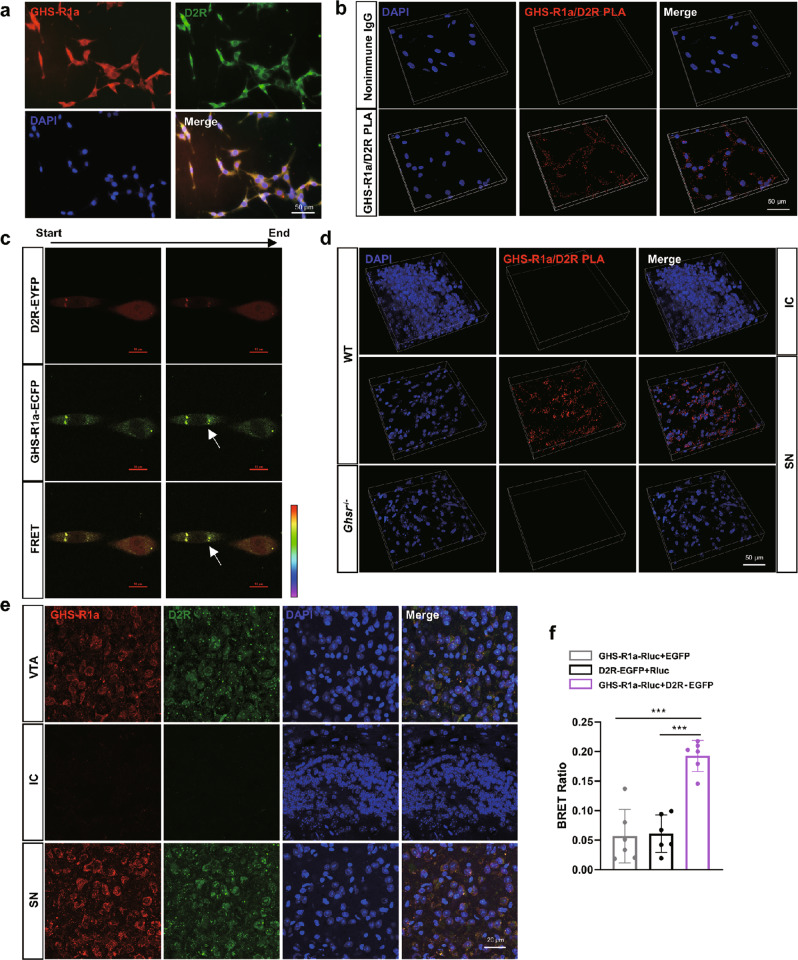
GHS-R1a and D2R formed heterodimers in vitro and in vivo. **a** Representative images for GHS-R1a and D_2_R co-expressing in PC-12 cells; **b** Measurement of PLA-positive dots for GHS-R1a/D_2_R heterodimers in PC-12 cells; **c** Representative confocal microscopy images for D_2_R-EYFP (top, red), GHS-R1a-ECFP (middle, green) and FRET pseudo-color ratio (GHS-R1a-ECFP/D_2_R-EYFP) (bottom), arrows indicate FRET imaging; **d** Measurement of PLA-positive dots for GHS-R1a/D_2_R heterodimers in the SN and IC of mice; **e** Identification of neurons co-expressing GHS-R1a and D_2_R in the SN, IC and VTA of mice; **f** Heterodimers of GHS-R1a and D_2_R were measured by BRET. Data are depicted as bar graphs with mean ± SEM. ***P < 0.001, *n* = 6.

We assessed the coexpression of GHS-R1a and D_2_R in PC-12 cells and the interactions between these two receptors. Immunofluorescence staining revealed that GHS-R1a and D_2_R were colocalized in PC-12 cells (Fig. 1a). To verify the interaction between GHS-R1a and D_2_R, we labeled GHS-R1a and D_2_R with specific antibodies and conducted PLA (Fig. 1b), which is a sensitive method to visualize and quantify protein interactions in situ [51–53]. FRET and BRET analyses were also performed to assess the interaction of GHS-R1a and D_2_R in PC-12 cells. In the FRET assay, PC-12 cells were transfected with GHS-R1a-ECFP and D_2_R-EYFP, and the changes in the FRET ratio were measured to assess the interaction of GHS-R1a between D_2_R. In Fig. 1c, green represents sites of GHS-R1a and D_2_R interaction, and the arrows indicate FRET. We were able to detect changes in the FRET ratio in PC-12 cells expressing GHS-R1a-ECFP and D_2_R-EYFP. For the BRET assay, similar to the FRET assay, PC-12 cells were transfected with GHS-R1a-Rluc and D_2_R-EGFP, and Western blotting showed that GHS-R1a and D_2_R protein levels were significantly increased after transfection (Supplementary Fig. 1). Moreover, the BRET ratio was measured after the addition of coelenterazine. Compared with those of negative control cells transfected with GHS-R1a-Rluc + EGFP and D_2_R-EGFP + Rluc, the BRET ratio of PC-12 cells cotransfected with D_2_R-EGFP and GHS-R1a-Rluc were increased by 240% (*P* < 0.001) and 220% (*P* < 0.001), respectively (Fig. 1f). These results indicate that GHS-R1a and D_2_R could form heterodimers in PC-12 cells.

We also assessed the colocalization of GHS-R1a and D_2_R and GHS-R1a/D_2_R heterodimers levels in the SN in WT mice (Fig. 1d, e). Due to the absence of D_2_R expression in the WT mice [26], the islands of calleja (IC) were selected as negative controls. Since D_2_R is highly expressed in the VTA [54, 55], we also tested the expression of D_2_R in the VTA to verify the specificity of D_2_R antibodies (Fig. 1e). Interactions between GHS-R1a and D_2_R could not be observed in the IC in WT mice or the SN in *Ghsr*^−/−^ mice by PLA (Fig. 1d, e). These results suggest that GHS-R1a and D_2_R were coexpressed in PC-12 cells as well as in dopaminergic neurons in the SN, where the two receptors could form heterodimers both in vitro and in vivo.

### The GHS-R1a and D_2_R interaction was weakened in MPP^+^/MPTP-treated cells

**Fig. 2:**
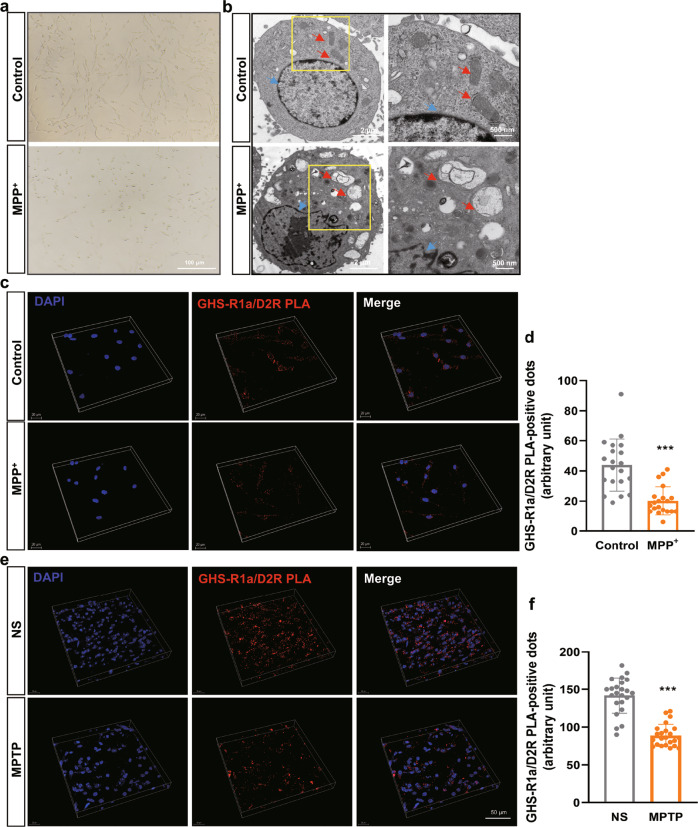
Effect of MPP^+^ and MPTP on the interaction of GHS-R1a and D2R in vitro and in vivo. **a** The decrease of PC-12 cell density induced by MPP^+^ was observed by inverted fluorescence microscope; **b** The ultrastructure of PC-12 cells treated with MPP^+^ was observed by transmission electron microscope. Red arrows indicate mitochondria and blue arrows indicate the nuclear membrane; **c** Representative images of GHS-R1a/D_2_R PLA-positive dots in PC-12 cells of control and MPP^+^ group; **d** Analysis of GHS-R1a/D_2_R PLA-positive dots in the PC-12 cells treated with vehicle or 2 mM MPP^+^ for 24 h; **e** Representative images of GHS-R1a/D_2_R PLA-positive dots in WT mice and PD mice; **f** Analysis of GHS-R1a/D_2_R PLA-positive dots in the MPTP-treated PD mice. Data are depicted as bar graphs with as mean ± SEM. ***P < 0.001, *n* = 20.

To evaluate the effect of 1-methyl-4-phenylpyridinium ion (MPP^+^)/MPTP on the formation of GHS-R1a/D_2_R heterodimers, PC-12 cells and C57BL/6 mice were treated with MPP^+^ and MPTP, respectively. As shown in Fig. 2a, b, the number and morphology of PC-12 cells were changed, and shrinkage of the nuclear membrane and swelling of mitochondria were also observed after incubation with 2 mM MPP^+^ for 24 h. The results of morphological analysis indicated that PC-12 cells were injured by 2 mM MPP^+^. PLA showed that the number of PLA fluorescence signals was decreased by 54% in the group treated with 2 mM MPP^+^ compared with the control group (Fig. 2c, d, *P* < 0.001), indicating that the interaction between GHS-R1a and D_2_R was weakened in MPP^+^-treated PC-12 cells. GHS-R1a/D_2_R heterodimers were also observed in the SN in MPTP-induced PD mice model, and we detected a 36.2% reduction in the number of PLA fluorescence signals in the SN in PD mice (Fig. 2e, f, *P* < 0.001), compared with control mice, indicating that the interaction between GHS-R1a and D_2_R was weakened in MPTP-induced PD mice model. These results indicate that MPP^+^/MPTP had inhibitory effects on GHS-R1a/D_2_R heterodimers formation in vitro and in vivo.

### Activation of GHS-R1a/D_2_R heterodimers attenuated MPP^+^-induced cell injury

**Fig. 3:**
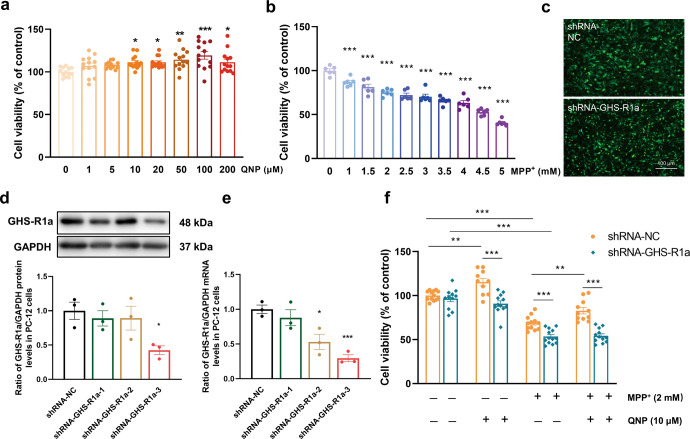
Effect of GHS-R1a/D_2_R heterodimers activation on cell viability. Effects of QNP (**a**) and MPP^+^ (**b**) on cell viability at indicated concentrations in PC-12 cells; **c** The efficiency of virus infection was detected by fluorescence microscope; **d**, **e** The protein and mRNA levels of GHS-R1a were decreased in shRNA-GHS-R1a-3 cells; **f** Pre-incubation with QNP (10 μM) antagonized MPP^+^ (2 mM)-induced reduction in cell viability in shRNA-NC group, as determined by CCK-8 assay. Data are depicted as bar graphs with mean ± SEM. *P < 0.05 vs. control, **P < 0.01 vs. control, ***P < 0.001 vs. control, *P < 0.05 vs. shRNA-NC, ***P < 0.001 vs. shRNA-NC, *n* = 12, 12, 3, 3, 12.

Then, we investigated whether activation of GHS-R1a/D_2_R heterodimers could affect MPP^+^-induced cell damage. The CCK-8 assay identified 10 μM and 2 mM as the effective concentrations of QNP and MPP^+^, respectively (Fig. 3a, b). As shown in Fig. 3f, cell viability was decreased by 31.3% (*P* < 0.001) and 44.7% (*P* < 0.001) in the shRNA-NC group and shRNA-GHS-R1a group, respectively, after MPP^+^ treatment. Compared with that in the shRNA-NC group, the cell viability in the shRNA-NC-QNP group was significantly increased by 15.3% (*P* = 0.002). However, compared with that in the shRNA-NC-QNP group, the cell viability in the shRNA-GHS-R1a-QNP group was decreased by 21.1% (*P* < 0.001). Compared with that in the shRNA-NC-QNP + MPP^+^ group, the cell viability in the shRNA-GHS-R1a-QNP + MPP^+^ group was decreased by 34.3% (*P* < 0.001). In addition, compared with that in the shRNA-NC-MPP^+^ group, the cell viability in the shRNA-NC-QNP + MPP^+^ group showed a 20.4% (*P* = 0.006) increase, which might be related to the activation of GHS-R1a/D_2_R heterodimers. These results suggest that QNP could activate the GHS-R1a/D_2_R heterodimers, exerting protective effects against cell death.

### Activation of GHS-R1a/D_2_R heterodimers improved motor function in MPTP-induced PD mice model

**Fig. 4:**
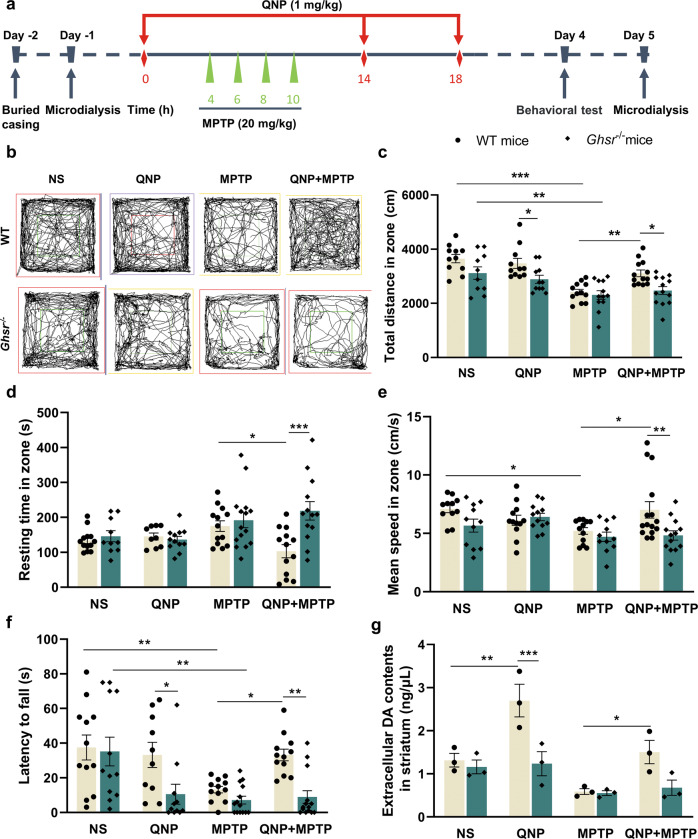
Effects of GHS-R1a/D_2_R heterodimers activation on motor abilities in MPTP-induced PD mice model. **a** Schematic diagram of animal modeling; **b** The trajectory of open field in different groups; **c**–**e** The distance traveled, resting time and mean speed in the open field were evaluated; **f** The residence time of mice in different groups on rotarod was assessed by the rotarod test; **g** Extracellular DA contents in the striatum were determined by microdialysis. Data are depicted as bar graphs with mean ± SEM, *P < 0.05, **P < 0.01, ***P < 0.001, *n* = 10–13, 3.

To determine the roles of GHS-R1a/D_2_R heterodimers in PD mice, mice were subjected to behavioral tests 0.5, 1, 2, 4 and 6 h after intraperitoneal injection of 0.5/1 mg/kg QNP [53, 56–58] (Supplementary Figs. 2–4). Ultimately, 4 h and 1 mg/kg were selected as the optimal treatment duration and concentration of QNP. In the open field experiment, we measured the total distance traveled, time spent in the zone and average speed of the mice. Our results showed that compared with the WT-NS and *Ghsr*^*−/−*^-NS groups, the WT-MPTP and *Ghsr*^*−/−*^-MPTP groups exhibited a 33.9% (*P* < 0.001) and 25.9% (*P* = 0.003) reduction in total distance traveled, respectively (Fig. 4c). Compared with that of the WT-NS and *Ghsr*^*−/−*^-NS groups, the mean speed of the WT-MPTP and *Ghsr*^*−/−*^-MPTP groups was decreased by 26.6% (*P* = 0.037) and 16.1%, respectively (Fig. 4e). The total distance traveled was decreased by 17.0% (*P* = 0.039) in the *Ghsr*^−/−^-QNP group compared with the WT-QNP group (Fig. 4c). The total distance traveled, the mean speed and the time spent in the zone were increased by 34.8% (*P* = 0.029), 28.8% (*P* = 0.008), and decreased by 41.4% (*P* = 0.023), respectively, in the WT-QNP + MPTP group compared with the WT-MPTP group. Compared with the WT-QNP + MPTP group, the *Ghsr*^−/−^-QNP + MPTP group exhibited a 20.5% (*P* = 0.011) reduction in total distance traveled, a 112.1% (*P* < 0.001) increase in the time spent in the zone, and a 31.4% (*P* = 0.003) decrease in mean speed (Fig. 4b–e).

Moreover, in the rotarod test, compared with the WT-NS and *Ghsr*^*−/−*^-NS groups, the WT-MPTP and *Ghsr*^*−/−*^-MPTP groups exhibited a 64.9% (*P* = 0.009) and 79.7% (*P* = 0.001) reduction in time spent on the rod, respectively (Fig. 4f). The time spent on the rod was increased by 151.9% (*P* = 0.043) in the WT-QNP + MPTP group compared with the WT-MPTP group. The time spent on the rod was decreased by 68.2% (*P* = 0.022) in the *Ghsr*^−/−^-QNP group compared with the WT-QNP group. The time spent on the rod was decreased by 73.1% (*P* = 0.004) in the *Ghsr*^*−/−*^-QNP + MPTP group compared with the WT-QNP + MPTP group (Fig. 4f). The above results suggest that activation of the GHS-R1a/D_2_R heterodimers by QNP could improve the motor function in MPTP-induced PD mice model.

Then, extracellular DA contents in the different groups were determined by microdialysis. Our results showed that extracellular DA contents were increased by 105.1% (*P* = 0.002) in the WT-QNP group compared with the WT-NS group. And the extracellular DA contents were 54.3% (*P* < 0.001) lower in the *Ghsr*^−/−^-QNP group than in the WT-QNP group and 155.1% (*P* = 0.041) higher in the WT-QNP + MPTP group than in the WT-MPTP group (Fig. 4g). The above results indicate that the improvement in the motor function of MPTP-treated PD mice model might have been associated with increased extracellular DA contents in the striatum.

### The CaM/CREB signaling pathway mediated the neuroprotective effect of the GHS-R1a/D_2_R heterodimers in PD

**Fig. 5:**
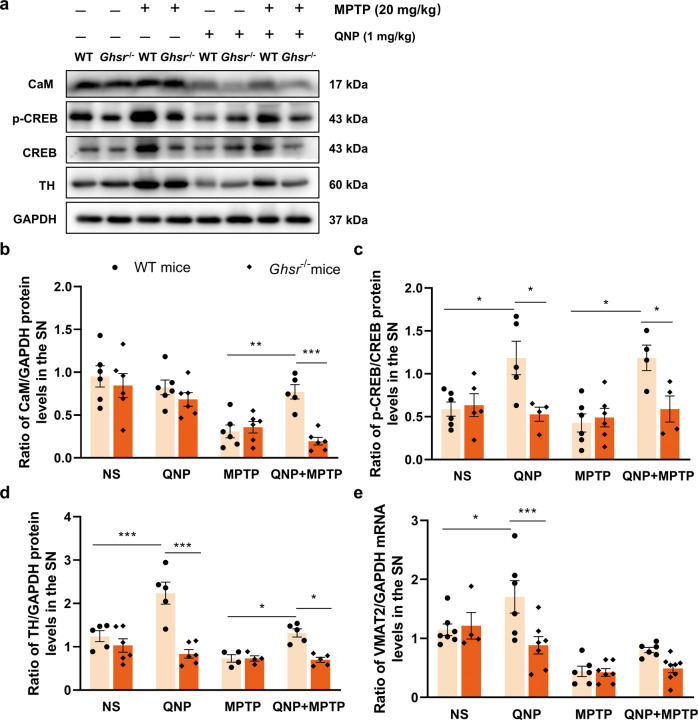
CaM/CREB pathway involved in the protection of dopaminergic neurons by GHS-R1a/D_2_R heterodimers activation. **a**–**d** Western blotting was applied to detect the levels of CaM, p-CREB, CREB and TH in the SN of WT and *Ghsr*^−/−^ mice treated with QNP and MPTP; **e** VMAT2 mRNA levels in the SN of WT and *Ghsr*^−/−^ mice treated with QNP and MPTP. Data are depicted as bar graphs with mean ± SEM, *P < 0.05, **P < 0.01, ***P < 0.001, *n* = 5.

To verify the mechanism underlying the increase in extracellular DA contents in the striatum induced by QNP, we assessed the expression of CaM/CREB pathway-related proteins and their downstream target proteins, TH and VMAT2. Our results showed that CaM protein levels in the SN were increased by 149% (*P* = 0.008) in the WT-QNP + MPTP group compared with the WT-MPTP group. CaM protein levels in the SN were decreased by 74.8% (*P* < 0.001) in the *Ghsr*^−/−^-QNP + MPTP group compared with the WT-QNP + MPTP group (Fig. 5a, b).

Then, we measured the protein levels of p-CREB and CREB. The p-CREB/CREB ratio in the SN was increased by 101.3% (*P* = 0.009) in the WT-QNP group compared with the WT-NS group. The p-CREB/CREB ratio in the SN was decreased by 55.4% (*P* = 0.007) in the *Ghsr*^−/−^-QNP group compared with the WT-QNP group. Moreover, the p-CREB/CREB ratio in the SN was increased by 177.4% (*P* = 0.002) in the WT-QNP + MPTP group compared with the WT-MPTP group. The p-CREB/CREB ratio in the SN was decreased by 50.3% (*P* = 0.024) in the *Ghsr*^−/−^-QNP + MPTP group compared with the WT-QNP + MPTP group (Fig. 5a, c). These results demonstrate that activation of the GHS-R1a/D_2_R heterodimers could increase CaM protein levels and the p-CREB/CREB ratio in the SN in MPTP-induced PD mice model.

Subsequently, we evaluated the expression of TH by Western blotting. The results showed that TH protein levels in the SN were increased by 79.8% (*P* < 0.001) in the WT-QNP group compared with the WT-NS group. Compared with the WT-QNP group, the *Ghsr*^−/−^-QNP group exhibited a 62.6% (*P* < 0.001) decrease in TH protein levels in the SN. Similarly, TH protein expression in the SN was 69.1% (*P* = 0.035) higher in the WT-QNP + MPTP group than in the WT-MPTP group. TH protein levels in the SN were decreased by 47% (*P* = 0.012) in the *Ghsr*^−/−^-QNP + MPTP group compared with the WT-QNP +MPTP group (Fig. 5a, d). These results suggest that activation of the GHS-R1a/D_2_R heterodimers promoted TH protein expression in the SN in MPTP-induced PD mice model.

Finally, the changes in VMAT2 mRNA levels were measured by RT-PCR, and the results are shown in Fig. 5e. VMAT2 mRNA expression in the SN was increased by 48.8% (*P* = 0.025) in the WT-QNP group compared with the WT-NS group, while compared with that in the WT-QNP group, VMAT2 mRNA expression in the *Ghsr*^−/−^-QNP group was decreased by 48.2% (*P* < 0.001). These results indicate that the activation of GHS-R1a/D_2_R heterodimers could promote the transcription of the CREB downstream target VMAT2 but did not affect the mRNA expression of VMAT2 in the SN of MPTP-induced PD mice model.

## Discussion

**Fig. 6:**
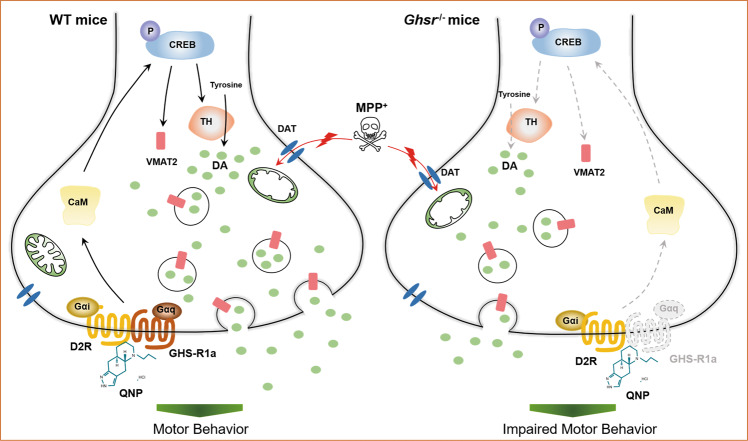
The role of GHS-R1a/D_2_R heterodimers activation in dopaminergic neurons. GHS-R1a and D_2_R receptors form heterodimers in dopaminergic neurons, GHS-R1a/D_2_R heterodimers activation upregulates TH expression through CaM-mediated CREB signaling pathway, promotes synthesis and release of DA, and antagonizes MPTP-induced behavioral changes in WT mice.

In the present study, we demonstrated that endogenous GHS-R1a and D_2_R could form heterodimers both in vitro and in vivo and that GHS-R1a/D_2_R heterodimers formation was reduced in MPTP-induced PD mice model. Activation of GHS-R1a/D_2_R heterodimers by QNP conferred neuroprotection in PD mice by promoting DA synthesis and release. The underlying mechanisms might be related to the elevation of CaM and p-CREB protein levels, resulting in enhanced TH expression (Fig. 6).

Several recent studies have shown that GHS-R1a is able to form heterodimers with two types of dopamine receptors, D_1_R and D_2_R [23, 59–61]. In the brain, D_1_R is highly expressed in the striatum, nucleus accumbens, olfactory bulb, amygdala and frontal cortex, while D_2_R is mainly expressed in the SN, striatum, nucleus accumbens, olfactory tubercle, VTA, hypothalamus and hippocampus [62, 63]. D_2_R, but not D_1_R, is expressed in the presynaptic membranes of dopaminergic neurons in the SN [64–69], where GHS-R1a is also expressed [60, 70, 71]. This provides a basis for the formation of the GHS-R1a/D_2_R heterodimers in dopaminergic neurons in the SN. In our study, for the first time, we observed that GHS-R1a and D_2_R could form heterodimers in PC-12 cells and dopaminergic neurons.

To verify the function of GHS-R1a/D_2_R heterodimers in PD, QNP was used to induce the activation of GHS-R1a/D_2_R heterodimers [59, 60, 72]. In vitro, we observed that QNP alone enhanced cell viability in the shRNA-NC group and MPP^+^-treated shRNA-NC group but not in the shRNA-GHS-R1a group. Furthermore, some studies have also shown that the length of primary dendrites, number of dendrites and cell body area of midbrain dopaminergic neurons derived from human-induced pluripotent stem cells were significantly increased after QNP treatment, and these phenomena may be related to the PI3K-ERK1/2 signaling pathway [73]. In vivo, we observed that QNP could increase the total distance traveled and mean speed in the open field test and time spent on the rod in the rotarod test or even reverse the decreases in these parameters caused by MPTP in WT mice. Moreover, we found that the extracellular DA contents in MPTP-treated WT mice were increased by QNP treatment; however, none of these changes were observed in GHS-R1a knockout mice. These results indicated that activation of GHS-R1a/D_2_R heterodimers by QNP could increase extracellular DA contents in the striatum, ultimately improving motor performance in MPTP-induced PD mice model. Similarly, Shao et al. found that repeated QNP treatment significantly reduced SN inflammation and resisted MPTP-induced dopaminergic neuron damage [74], but the mechanisms underlying the effects of QNP have not been elucidated.

In the nigral dopaminergic neurons of PD patients, neuroplasticity and axonal and synaptic plasticity can be regulated by the CREB signaling pathway [36]. Inhibition of CREB activation in vitro leads to neuronal differentiation impairment. The survival rate of newborn neurons in transgenic mice with CREB deficiency is low [34]. This evidence suggests that the CREB pathway is of great importance in maintaining normal nervous system function. In our study, we observed that the expression of CaM and the p-CREB/CREB ratio in the SN were decreased in MPTP-induced PD mice model. Activation of GHS-R1a/D_2_R heterodimers by QNP could promote CaM-mediated CREB phosphorylation. CREB is involved in the formation of DNA/protein complexes between the cAMP response element and the TH and VMAT2 genes and, together with CREB-binding protein, regulates the transcription of genes [46, 75]. Then, we investigated the effect of GHS-R1a/D_2_R heterodimers activation on VMAT2 and TH expression in MPTP-induced PD mice model.

VMAT2, one of the downstream targets of CREB, is responsible for mediating the release of DA from the presynaptic membrane [76–79]. In our study, we did not observe any significant changes in VMAT2 mRNA levels after QNP treatment. TH is the rate-limiting enzyme of DA synthesis by dopaminergic neurons [80] and plays an important role in DA biosynthesis. TH can be used as a marker of dopaminergic neurons, and the pathogenesis of PD is related to a reduction in TH levels [81]. Previous studies have shown that the mRNA and protein levels of TH in the SN and TH enzyme activity were decreased in PD animal models [82–85]. Relevant clinical experiments have also indicated that TH gene and protein levels and activity in the SN exhibit abnormal changes in PD patients [86], leading to the clinical symptoms of the disease. Injection of TH-transfected nerve cells into the striata of PD rats can relieve PD-related symptoms [87]. Moreover, delivery of human-derived TH into the striata of PD rats can ameliorate PD symptoms, enhance TH activity, increase DA synthesis and alleviate abnormal rotation behavior [88, 89]. We observed that TH protein expression was significantly upregulated in WT mice and MPTP-induced PD mice model after QNP treatment. These results indicated that GHS-R1a/D_2_R heterodimers activation could promote TH expression but not VMAT2 expression through the CaM/CREB pathway. TH could promote DA synthesis, increase extracellular DA contents in the striatum, and ultimately promote motor function improvement in animals. However, this selective regulation of VMAT2 and TH expression may be related to the preferential nature of the regulatory effect of CREB after activation by GHS-R1a/D_2_R heterodimers [90–92], and whether other regulatory mechanisms exist remains to be elucidated.

In conclusion, GHS-R1a and D_2_R were expressed and could form heterodimers in dopaminergic cells and neurons in the SN. Activation of GHS-R1a/D_2_R heterodimers by QNP significantly improved the motor function of MPTP-induced PD mice, and the mechanism might be related to GHS-R1a/D_2_R heterodimers activation, which regulates the CaM/CREB pathway, promotes the synthesis and release of DA by upregulating TH expression, and ultimately alleviates MPTP-induced injury in mice. Considering that GHS-R1a is highly expressed in the SN, has high constitutive activity, and dimerizes with other receptors, the present study might provide new targets for the pharmacological treatment of PD.

## Supplementary information

Supplementary Figure 1

Supplementary Figure 2

Supplementary Figure 3

Supplementary Figure 4

Supplementary Figure Legend

## Data Availability

All data generated or analyzed during this study are included in this published article and its Supplementary files.
