# Supplementary figures and images for: Quinpirole ameliorates nigral dopaminergic neuron damage in Parkinson’s disease mouse model through activating GHS-R1a/D2R heterodimers
Source: Acta Pharmacol Sin. 2023 Mar 10;44(8):1564–75. doi: 10.1038/s41401-023-01063-0 (PMC10374575; doi:10.1038/s41401-023-01063-0)

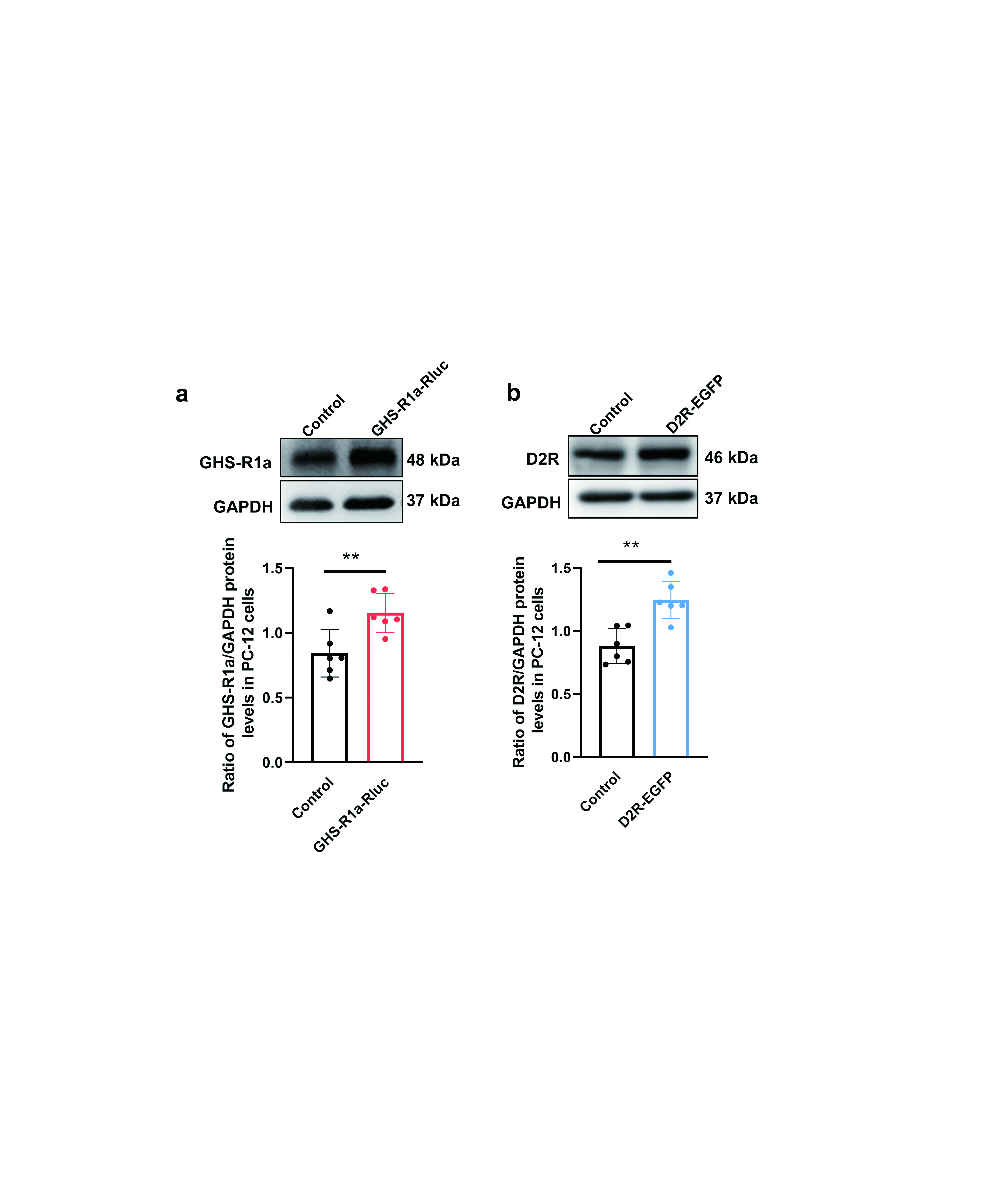

Supplement: Supplementary file 1 — Supplementary Figure 1 [file 41401_2023_1063_MOESM1_ESM.tif]

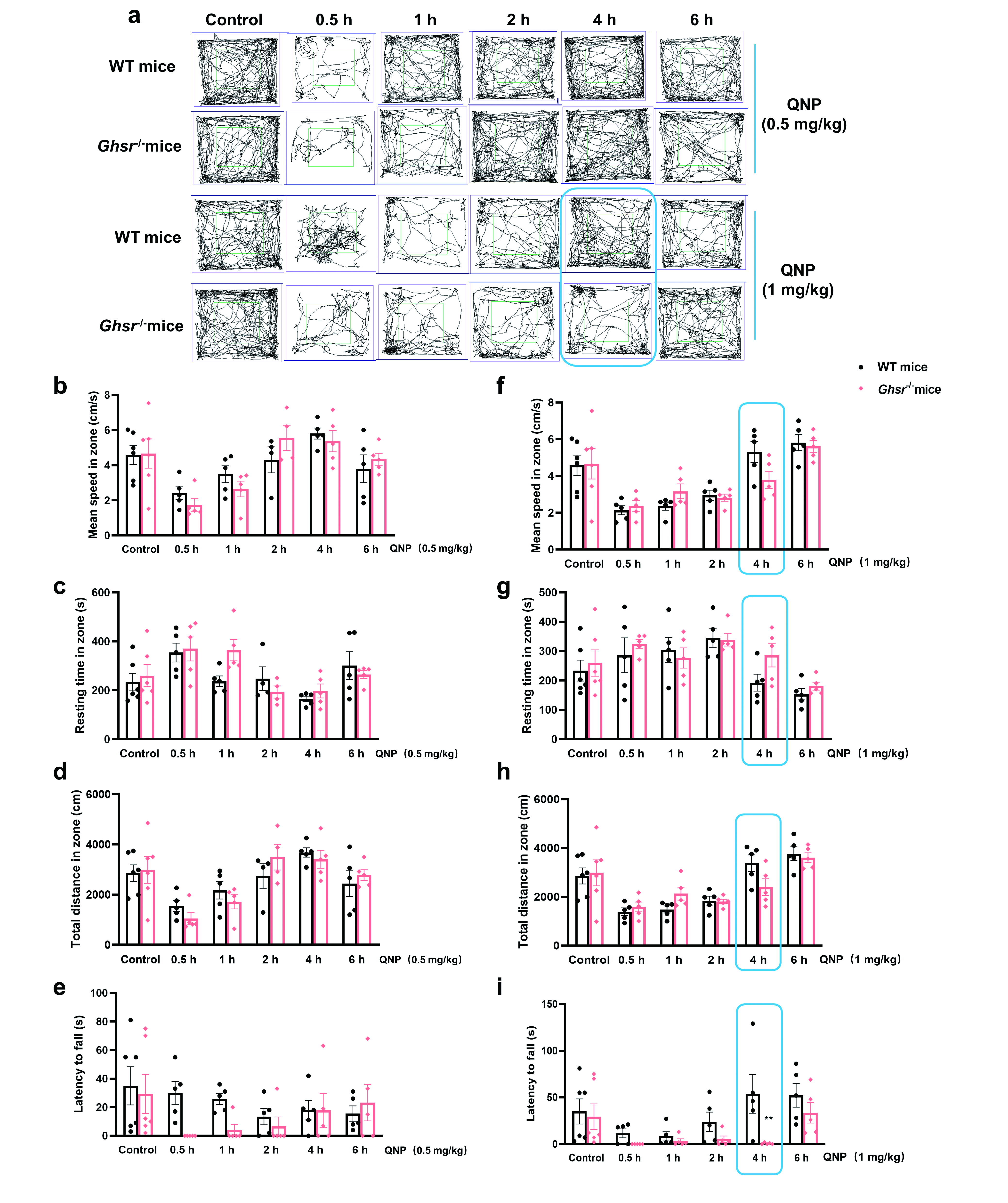

Supplement: Supplementary file 2 — Supplementary Figure 2 [file 41401_2023_1063_MOESM2_ESM.tif]

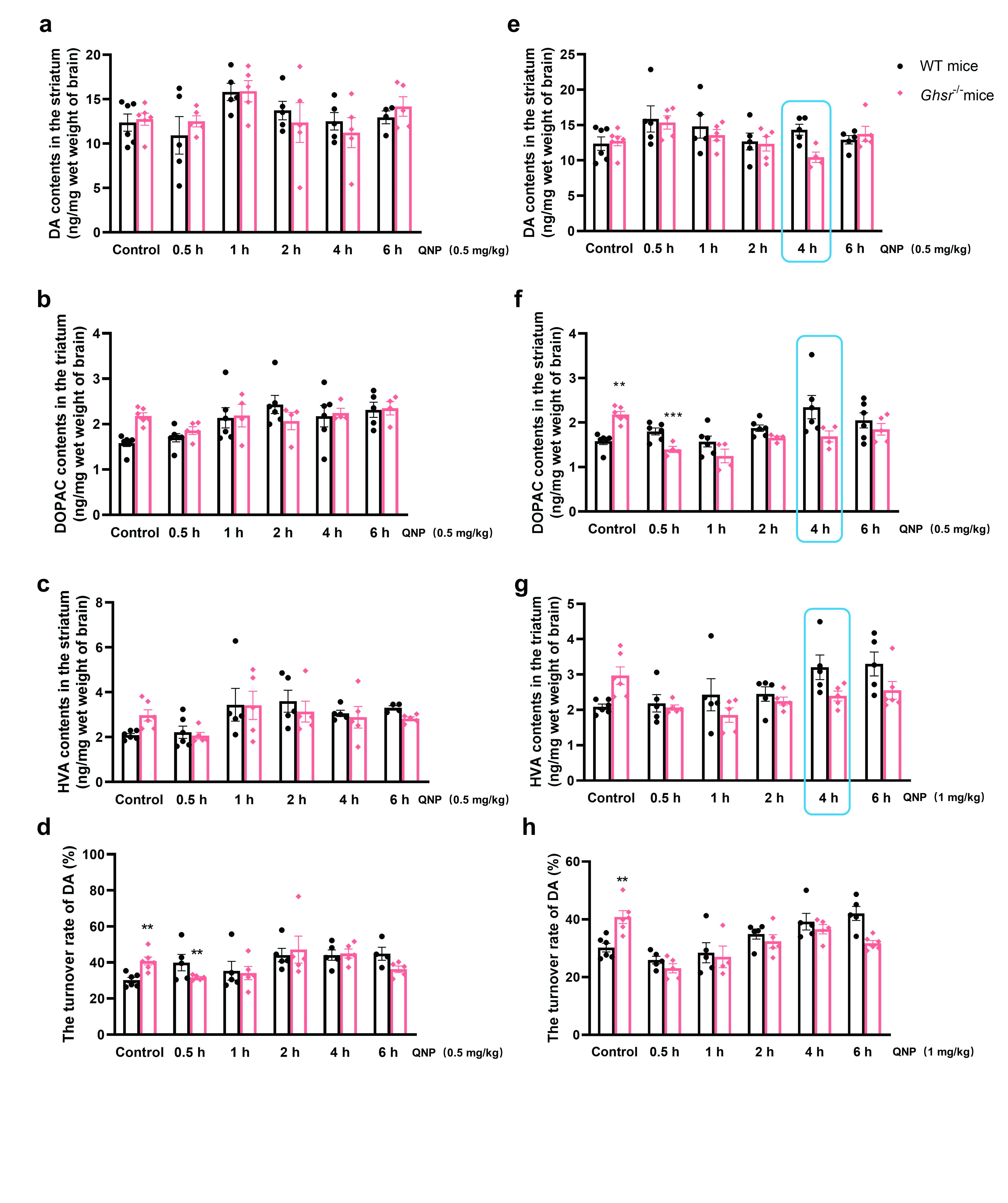

Supplement: Supplementary file 3 — Supplementary Figure 3 [file 41401_2023_1063_MOESM3_ESM.tif]

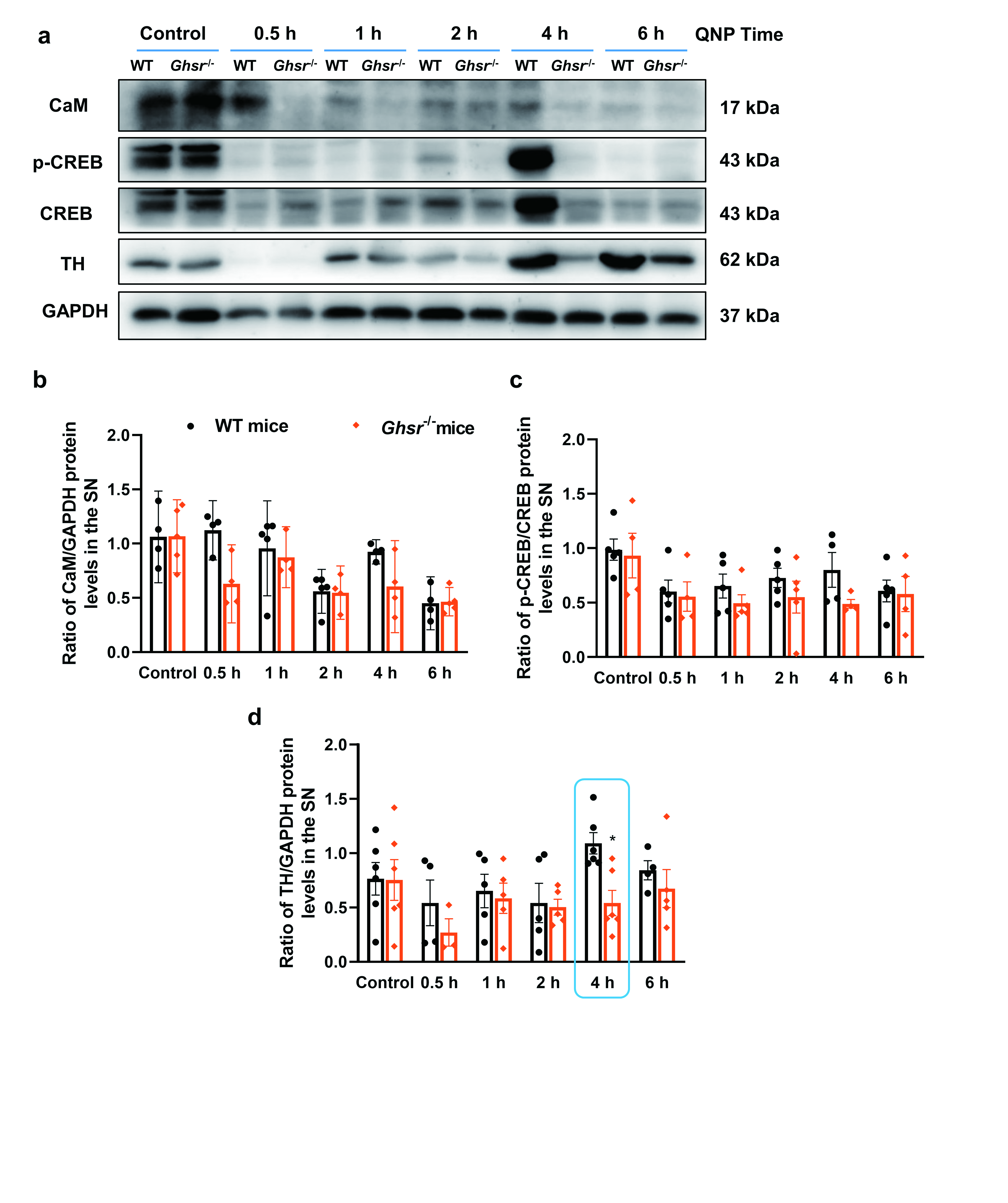

Supplement: Supplementary file 4 — Supplementary Figure 4 [file 41401_2023_1063_MOESM4_ESM.tif]
