# Supplementary material for: Quinpirole ameliorates nigral dopaminergic neuron damage in Parkinson’s disease mouse model through activating GHS-R1a/D2R heterodimers
Source: Acta Pharmacol Sin. 2023 Mar 10;44(8):1564–75. doi: 10.1038/s41401-023-01063-0 (PMC10374575; doi:10.1038/s41401-023-01063-0)
Supplement: Supplementary file 5 — Supplementary Figure Legend [file 41401_2023_1063_MOESM5_ESM.docx]

**S-Fig. 1 The changes of GHS-R1a and D_2_R protein levels in PC-12 cells were detected by western blotting.** The GHS-R1a (a) and D_2_R (b) protein levels of GHS-R1a-Rluc and D_2_R-EGFP groups were significantly increased respectively, compared with control group. Data are depicted as bar graphs with mean ± SEM. *^**^P*<0.01, n=6.

**S-Fig. 2 Effects of different concentrations of QNP on locomotor activity in WT and *Ghsr*^-/-^ mice.** (a) The trajectory of open field in WT and *Ghsr*^-/-^ mice which were given 0.5 or 1 mg/kg QNP at 0.5 h, 1 h, 2 h, 4 h and 6 h; (b-d) The mean speed, resting time and distance travelled in the open field in 0.5 mg/kg QNP were evaluated; (f-h) The mean speed, resting time and distance travelled in the open field in 1mg/kg QNP were evaluated; The residence time of mice in different groups on rotarod treadmills was assessed by the rotarod test in 0.5 (e) and 1 (i) mg/kg QNP. Data are depicted as bar graphs with mean ± SEM. ^**^*P*<0.01, n=5.

**S-Fig. 3 Effects of different concentrations of QNP on DA levels and its metabolites in striatum in WT and *Ghsr*^-/-^ mice.** Striatal DA (a), DOPAC (b), HVA (c) levels and the turnover rate of DA by HPLC-ECD (d) in mice after QNP concentration of 0.5 mg/kg; Striatal DA (e), DOPAC (f), HVA (g) levels and the turnover rate of DA by HPLC-ECD (h) in mice after QNP concentration of 1 mg/kg. Data are depicted as bar graphs with mean ± SEM. n=5.

**S-Fig. 4 The changes of CaM, TH, p-CREB and CREB protein levels in the SN of WT and *Ghsr*^-/-^ mice induced by QNP.** (a) After 0.5 h, 1 h, 2 h, 4 h and 6 h of QNP treatment, the changes of CaM, p-CREB, CREB and TH protein levels in the SN of WT and *Ghsr*^-/-^ mice induced by 1mg/kg QNP; (b-d) Statistical analysis. Data are depicted as bar graphs with mean ± SEM. ^*^*P*<0.05, n=5.
